# The Priority Goals and Underlying Impairments Contributing to Goal-Related Problems of People with Parkinson's Disease Receiving a Community-Based Rehabilitation Program

**DOI:** 10.1155/2024/9465326

**Published:** 2024-04-12

**Authors:** Sarah J. Davies, Hannah L. Gullo, Emmah Doig

**Affiliations:** ^1^School of Health and Rehabilitation Sciences, Faculty of Health and Behavioural Sciences, The University of Queensland, Brisbane, QLD 4072, Australia; ^2^School of Health, University of the Sunshine Coast, Sunshine Coast, Queensland 4556, Australia; ^3^Allied Health Research Collaborative, The Prince Charles Hospital, Chermside, QLD 4032, Australia; ^4^Surgical Treatment and Rehabilitation Service (STARS) Education and Research Alliance, The University of Queensland and Metro North Health, Brisbane, Australia

## Abstract

**Background:**

Goal setting is a core rehabilitation practice in Parkinson's disease (PD). Targeting therapy towards specific goals leads to greater improvements in performance and psychosocial outcomes. Goal setting in PD is feasible, and although the nature of goals has been described in previous studies, the underlying impairments related to goals have not been described. Understanding the nature of goals ensures that interventions for people with PD are aligned with their needs and priorities. Understanding the underlying impairments highlights which symptoms have the biggest impact on daily life and is necessary for planning appropriate interventions to target them.

**Aim:**

To describe the nature of the goals of people with PD; the underlying impairments related to goals; and to compare differences between high and low priority goals.

**Method:**

Deductive content analysis was used to map goal statements to the international classification of function (ICF) activity and participation category and to map therapist field notes detailing the primary underlying impairment to the ICF Body Functions category. These results were then compared across goal priority rankings.

**Results:**

88 goals of 22 people with PD were analysed. We found that people with PD set diverse goals across all chapters of the ICF Activity and Participation category, with “self-care” goals making up the highest proportion of goals. The primary underlying impairment related to the goals was predominantly related to impairments in “mental functions” under the Body Functions category. Regardless of goal priority, most goal-related underlying impairments were found to be in the “mental functions” category.

**Conclusion:**

The goals of this sample of community-dwelling people with PD highlight their diverse needs and priorities. These findings indicate that nonmotor symptoms, namely, executive dysfunction and amotivation most commonly impact the performance of and participation in activities of greatest importance to people with PD. This trial is registered with ACTRN12621001483842.

## 1. Introduction

Rehabilitation interventions are ideally person-centred and tailored to individual needs; consequently, person-centred goal-setting is a core recommended practice [1]. This is evidenced by its inclusion in clinical guidelines [2] and international recommendations [3, 4] for neurorehabilitation. Goals direct efforts toward activities that are relevant and meaningful to the person, motivating participation during rehabilitation, driving persistence, and use of task-relevant knowledge and strategies [5]. Both goals and a person's engagement in goal-setting are important parts of rehabilitation interventions [6]. Research has shown that targeting therapy toward specific goals leads to greater changes in performance for people with Parkinson's disease (PwPD) than standardised, impairment-focused approaches [7]. According to a Cochrane review of goal setting in rehabilitation for adults with acquired disability, the best evidence indicated that goal setting may have positive effects on psychosocial outcomes, such as quality of life, emotional status, and self-efficacy [8]. This is particularly important in Parkinson's disease (PD), as the burden of the disease is large and its impact on psychosocial outcomes is well documented [9].

Goal setting is the most frequently reported psychosocial intervention used by occupational therapists working with PwPD [10]. Goal-setting in PD has been the focus of several studies in recent years [11–14]. These studies have primarily focused on the feasibility of person-centred goal setting in the presence of cognitive impairment and the nature of the goals set by PwPD. Kang et al. [11] and Watermeyer et al. [14] concluded that goal setting is feasible for PwPD who have subjective cognitive complaints and early-stage PD dementia. Despite the difference in the severity of cognitive impairments between these sample populations, common categories of goals were evident, including disease/symptom management; executive functioning (planning and organising); time management; memory; technology; leisure participation; home establishment/management; and medication management. Vlagsma et al. [13] compared the goals and cognitive profiles of PwPD and people with acquired brain injuries and found similar goals and profiles in both neurological populations. The nature of the goals identified in Vlagsma et al.'s [13] study was also similar to the above-stated studies involving PwPD with subjective cognitive complaints and early-stage PD dementia.

Literature reports that person-centred goals are more closely aligned with people's expressed needs and desires than clinician-led goals [15]. This may be because person-centred goal setting takes a holistic view, addressing the person's difficulties in everyday life, considering them as an expert, and respecting the person “behind” the impairment or disease [16]. From this perspective, person-centred goal setting can offer insights into the perceptions of PwPD about their motivations, perceived burden of PD symptoms, and priorities for rehabilitation. Literature suggests that nonmotor symptoms of fatigue, psychological health, sleep, and cognitive functions are common concerns from the earliest stages of PD [17] and that addressing nonmotor symptoms in research is a priority for PwPD [18]. While the concerns and priorities for PwPD have been explored, there is a gap in understanding which symptoms or underlying impairments affect goal-related performance for PwPD receiving community rehabilitation. Similarly, although there is established evidence for the feasibility and importance of person-centred goal setting in PD, there is a gap in understanding the nature of goals outside of cognitive rehabilitation programs. To investigate these gaps, we conducted a study to explore the nature of the goals identified in a sample of PwPD who were participating in an intervention feasibility trial. The trial aimed to investigate the efficacy and feasibility of a novel intervention which included the use of person-centred goal-setting in a sample of community dwelling PwPD. The aims of this study were to

1. Describe the goals of PwPD using the International Classification of Function (ICF);
2. Identify the primary underlying impairment related to goal selection using the ICF;
3. Explore whether high- and low-priority goals differed according to goal type and related underlying impairment.

## 2. Method

### 2.1. Design

The study is a nested cohort study that describes the goals and underlying PD-related impairments of a sample of participants who were involved in a parallel group, randomised controlled (RCT) feasibility trial [19]. The RCT explored the feasibility and efficacy of the cognitive orientation to daily occupational performance (CO-OP) approach for PwPD. CO-OP is “a client-centred, performance-based, problem-solving approach that enables skill acquisition through a process of strategy use and guided discovery” ([20], p.2). CO-OP is a structured, manualised, and widely established approach with evidence of its efficacy across multiple studies and populations, including those with acquired brain injury, stroke, subjective cognitive complaints, mild dementia, spina bifida, and cerebral palsy [21–27]. To the best of our knowledge, this is the first study to trial the application of the CO-OP approach with a sample of PwPD.

All participants underwent a goal-setting session to identify their goals, which were targeted using the CO-OP intervention. The individualised CO-OP intervention was delivered face-to-face by a CO-OP-certified practitioner (SD) in the participants' homes. The intervention is described in detail elsewhere in Davies et al. [19]. Written informed consent was obtained from all participants prior to their entry into the trial. The study was approved by the UQ Human Research Ethics Committee (HREC)–2020/HE002650 on 9 February 2021, and the trial was registered with the Australian New Zealand Clinical Trials Registry, ACTRN12621001483842.

### 2.2. Participants

Participants were recruited using convenience sampling via Parkinson's Queensland Incorporated. PwPD were recruited to the study based on the following inclusion criteria: (a) aged over 18 years; (b) able to communicate in English; (c) able to provide informed consent; (d) living in the community; (e) availability of and experience using computer or tablet technology with Internet access; (f) willing to receive the intervention in their homes. Exclusion criteria were as follows: (a) premorbid or current major psychiatric or other neurological disorder; (b) diagnosis of dementia or severe cognitive impairment indicated by a score of less than 13 on the telephone version of the Montreal Cognitive Assessment; (c) significant sensory impairment (visual or hearing) which prevents involvement in the intervention and/or reliable completion of outcome measures; (d) communication disorder (severe expressive and/or receptive aphasia) which prevents involvement in the intervention and/or reliable completion of outcome measures; (e) complete dependence on others for personal care; and (f) receiving concurrent allied health or cognitive intervention working on the same goals.

### 2.3. Measures

Person-centred, occupation-based goals were identified using the Canadian Occupational Performance Measure (COPM), and the resultant goals were documented in objective, measurable terms, using the Goal Attainment Scale (GAS). The procedure for the combined use of COPM and GAS is from previous studies [28–30].

The COPM was used to identify and prioritise occupational performance goals in domains of self-care, productivity, and leisure [31]. It is widely used by occupational therapists with PwPD in clinical and research settings [32]. The COPM enables participants to rate the importance of their goals, perceived performance, and satisfaction with performance in goal areas, enabling goals to be prioritised and changes in self-rated performance to be measured. The COPM was used in this study to identify the participant's goals for their intervention and help rank each of the goals from highest to lowest priority.

GAS [33] is an individualised outcome measure widely used in neurological rehabilitation as a personalised and sensitive means of measuring goal attainment [34]. GASs are scaled using a 5-point measurement scale ranging from −2 to +2, with baseline performance scaled at −1 and expected outcomes scaled at zero. −2 captures the potential for deterioration, and +1 and +2 levels capture the potential for exceeding expected outcomes. The GAS was used in this study to operationalise and scale the goals identified by participants objectively and to document the expected level of achievement by program end.

Dynamic performance analysis (DPA) is a key feature of the CO-OP approach. The purpose of DPA is to resolve performance issues by identifying performance breakdowns and trialling solutions [35]. It is a dynamic, iterative process carried out by the CO-OP therapist, integrating task knowledge, occupational therapy theory, and clinical reasoning to identify effective and ineffective aspects of task performance. As DPA is focused on the specific way that an individual performs a specific activity, optimal performance is not assumed to be the result of a predefined, single sequence of actions but is individualised to the person and their abilities instead. The first stage of using DPA involved assessing whether participants were motivated and knew how to complete the goal-related activities (performer prerequisites). Then the participant was observed or described performing their goal-related activity to identify where and why breakdowns in performance were occurring (performance requisites). The verbal process of guided discovery was then implemented to help participants to “discover” ineffective aspects of task performance and develop strategies to solve them. The participant-derived strategies were then implemented therapeutically, and participants were taught to evaluate their performance to “check” which strategies were effective. Ineffective strategies were discarded, and the process was repeated until an effective strategy was discovered that enabled performance. The effective strategy was then recorded in the therapist's field notes as the outcome of the DPA. Examples of the DPA process are presented in Table 1.

Demographic information collected from participants included age, sex, disease duration, highest level of formal education, and work status. Several measures were administered on entry to the study to profile the sample in the areas of cognition, perceived cognition, activities of daily living (ADL), PD staging, and premorbid intelligence. These measures included:

Addenbrooke's Cognitive Evaluation-III (ACE-III; [36]) was used to measure participants cognitive status. The ACE-III is a brief cognitive screening battery assessing five neuropsychological domains (orientation and attention, memory, verbal fluency, language, and visuospatial function). Senda et al. [37] found that the ACE-III was a useful instrument to detect MCI based on the following cut-off scores: 100−89 indicating no cognitive impairment, 88−77, indicating MCI.

Perceived Deficits Questionnaire (PDQ; [38]) was used to measure participants perceived cognitive status. The PDQ is a self-rated 20-item tool assessing perceived cognitive functioning in four domains (attention, retrospective memory, prospective memory, and planning/organisation). Each item is rated on a 5-point scale from 0 (never) to 5 (almost always). A summary score ranging from 0–80 is generated with a higher score indicating greater perceived cognitive impairment.

The Nottingham Extended Activities of Daily Living scale (NEADL; [39]) was used to measure ADL status of participants. The NEADL consists of 22 items in four subscales (mobility, domestic, kitchen, and leisure). A summary score ranging from 0–22 is generated with higher scores, indicating greater independence. The items are rated using a 4-point Likert scale, scored from 0 (not at all, with help) to 1 (on my own with difficulty, on my own) in items representing general ADL tasks, including instrumental ADLs.

A modified Hoehn and Yahr (H&Y) scale [40] was used to measure disease severity. The H&Y scale is a 7-point Likert scale that focuses on the functional disability associated with PD. It identifies the progression of the disease through various stages from 1 (unilateral involvement only) to 5 (wheelchair bound or bedridden unless aided). Higher scores indicate more advanced PD.

The National Adult Reading Test (NART; [41]) was used to estimate the premorbid intelligence levels of participants. The NART is a 50-word reading test that provides reliable and valid estimates of intelligence quotient (IQ) and has been shown to be relatively insensitive to the effects of various neurological conditions, including PD [42]. All 50 words violate grapheme-phoneme correspondence rules (e.g., chord) thus testing vocabulary. Published equations were used to convert raw NART scores to predicted IQ scores on the Wechsler Adult Intelligence Scale.

### 2.4. Procedures

#### 2.4.1. Goal Setting and Dynamic Performance Analysis

**Figure 1:**
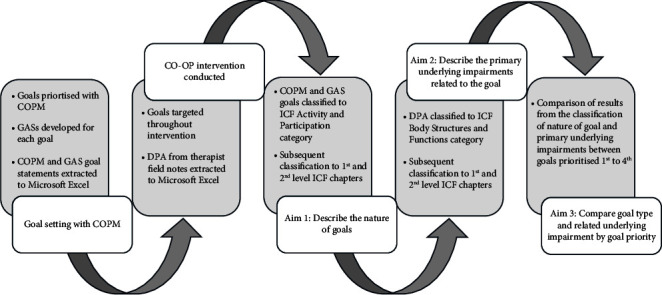
Study procedures flowchart.

The study procedures are overviewed in Figure 1. Prior to commencing the CO-OP intervention, participants each set four goals, with goal-setting conducted in participant's homes by SD using the COPM. Participants could select goals related to any challenges they perceived were most important for them to address; however, the COPM guides participants to develop occupation-focussed goals. Occupations are everything people do to occupy themselves, including looking after themselves (self-care), enjoying life (leisure), and contributing to the social and economic fabric of their communities (productivity; [43]. p. 34). Once goals were established, participants were asked to rank their goals from highest to lowest priority. Participants' three highest priority goals were targeted in the CO-OP intervention, and the fourth goal was not targeted in the intervention. The RCT was designed this way to enable measurement of transfer and generalisation of CO-OP to an “untrained goal.” Participants were made aware that the fourth goal would not be targeted when prioritising their goals. They were given another opportunity to reprioritise their goals prior to commencing their CO-OP intervention to ensure their most important goals were targeted during the intervention. GASs were developed for each of the four goals identified.

All targeted goals were addressed in each therapy session with discussion or observation regarding performance break down of goal activities occurring, followed by “discovering” and testing out solutions, in line with CO-OP. The information regarding the performance requisites and successful solutions (plans) was recorded for each participant in the therapist field notes, alongside therapist observations from each session. This iterative process of DPA allowed PD-related impairments that were primarily impacting on goal-related activities to be determined and documented. As the fourth goal was not targeted in the intervention, DPA for this goal took place during the goal setting and baseline measurement of the goal activity, not throughout the intervention as with higher priority goals.

#### 2.4.2. Data Collection

Demographic data (age, sex, education, work status, PD duration) and select self-report measures (PDQ, NEADL) were collected online via the Qualtrics survey. The profiling measures for cognition, premorbid IQ and PD stage were collected by SD during the baseline assessment in participant's home environments. The GAS expected levels of attainment, accompanying COPM goal statements, and corresponding goal priority ranking (1, 2, 3, 4) for each participant's goals were extracted and organised using Microsoft Excel. The written content about each participant's DPA was also extracted from the therapist field notes and entered into Microsoft Excel. The COPM, GAS, DPA data, and goal priority rankings were the units of analysis for this study.

### 2.5. Data Analysis

Participant demographic data and baseline measures of cognition, functional status, PD stage, and premorbid intelligence were summarised using descriptive statistics. A deductive content analytic approach [44] was applied to classify the types of goals set by participants, using the ICF as a framework [45]. For both the nature of goals and underlying impairments, the number and percentage in each ICF domain were calculated and the frequency in each chapter was tabulated by ICF category. ICF category and chapter codes were displayed visually and tabulated by frequency. The data that compared goals by their priority was descriptively analysed by tabulating and visually representing the data for the nature of goal proportion and underlying impairment proportion by goal priority number.

**Figure 2:**
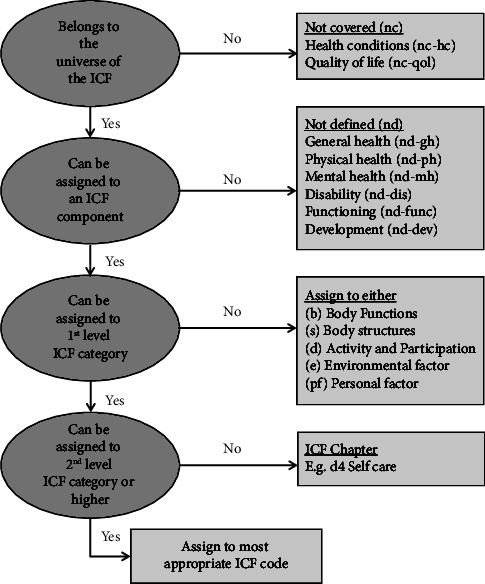
Goal coding system flow chart. Note: from Cieza et al. [46]. Refinements of the ICF linking rules to strengthen their potential for establishing comparability of health information. Disability and rehabilitation, 41 (5), 574–583. Reprinted with permission.

To address aim one, to describe the nature of the goals of PwPD, two members of the research team who were not involved with setting goals or delivering the intervention (HG and ED) first became familiar with the data by reading the extracted COPM goal and GAS 0 statements for each goal. Then a structured goal coding system was followed, based on the ICF and published linking rules (see Figure 2; [46]). HG and ED independently assigned a level one and two ICF chapter for each goal (using both the COPM and GAS statements). Once coding was completed, all authors held a series of consensus meetings to resolve any discrepancies.

To address aim two, to identify the primary underlying impairments related to the goals, the first author (SD) became familiar with the data by reading the extracted DPA statements. SD was the treating therapist in the trial who conducted goal-setting with participants and carried out the subsequent intervention, including the DPA. Using the same procedure described for aim one, SD assigned a level one and two ICF chapters for each goal using the extracted DPA statements. This analysis was carried out by SD as the knowledge of the participant's underlying impairments through observation during sessions, goal-setting conversations with participants about why they were having occupational performance problems (i.e., the PD-related impairments impacting on performance), as well as the documented DPA, was necessary to make a holistic judgement about the primary underlying PD-related impairments impacting performance.

To address aim three, to compare differences in goal types and goal-related impairments by goal priority number, we compared the results for goal type and underlying impairments between goals prioritised one to four.

## 3. Results

### 3.1. Participant Characteristics

22 participants were included in this study; the characteristics of the sample are outlined in Table 2. Participants were on average approximately five years postdiagnosis, most had tertiary level qualifications, and most were retired or not working due to their PD condition. The measure of functional independence (NEADL) broadly indicated that the sample had high levels of independence in everyday self-care and domestic activities. Most participants were stage 3 on the H&Y scale, indicating they had mild to moderate bilateral disease with some postural instability but were physically independent. When comparing the study sample with normative data for the ACE-III (cognitive status), 50% were below the norm for healthy controls. According to the diagnostic utility cut-off scores published by Senda et al. [37], two participants in the study met the criteria for MCI (scores 77–88) on the ACE-III. The PDQ attention and planning subscales had the highest mean scores at 8.00 and 7.27, respectively.

### 3.2. Nature of Goals

A total of 88 goals were extracted from the data set and included in the final analysis.  Table 3 shows goals grouped by ICF activities and participation category and chapter along with a description of the chapter and example goals. All nine first level chapters of the ICF activity and participation category were represented, with the largest proportion of goals categorised as “self-care” goals. The largest proportion of goals recorded were “managing diet and fitness” goals (*n* = 17) under “self-care.” The next highest proportion were goals related to “doing housework” (*n* = 8) under “domestic life,” “recreation and leisure” (*n* = 8) under “community, social, and civic life,” followed by “completing daily routines” (*n* = 7) under “general tasks and demands.”

### 3.3. Underlying Impairments Related to Goal Activities

Table 4 shows the goals grouped by ICF Body Functions category and chapter according to the primary underlying impairment, along with a description of the chapter and example goals. Three of the eight chapters of the ICF Body Functions category are present. The majority of goals (72.7%) had a primary underlying impairment related to “mental functions” and most of these were categorised as “planning and organisation” or “time management” goals. “Neuromusculoskeletal and movement-related functions” were the primary underlying impairment in 26% of goals and most of these goals were classified as “coordination of voluntary movements” and “control of complex voluntary movements” goals. There was a single “sensory functions and pain” goal in the sample. The other ICF Body Functions chapters are not represented.

### 3.4. Goal Type and Underlying Goal-Related Impairment According to Goal Ranking

For the third aim, we compared proportions of goal types and underlying impairments for PwPD's highest to lowest priority goals.  Table 5 indicates that goals, regardless of priority, were mostly related to impairments in “mental functions.”

Several ICF Activity and Participation chapters including “self-care,” “general tasks and demands,” “mobility,” “domestic life,” and “community, social and civic life” were represented across all goal numbers, as presented in Table 6. “Domestic life” goals were the largest proportion of lowest priority goals, whereas “self-care” goals were the largest proportion for higher priority goals.

## 4. Discussion

This study explored the nature of goals and underlying impairments contributing to goal-related problems of PwPD receiving a community-based rehabilitation program. The nature of goals across chapters of the ICF activity and participation category were diverse, and the goals were primarily related to impairments in “mental functions” under the ICF Body Functions category. Regardless of goal priority, most goals related to impairments in “mental functions,” with “self-care” goals making up the greatest proportion of people's higher priority goals, and “domestic life” goals making up the greatest proportion of their lowest priority goals.

Consistent with previous literature, we found that PwPD formulated rehabilitation goals to manage their functioning in daily life [11, 13, 14]. The common goal areas in all studies were “self-care,” “general tasks and demands,” “domestic life,” and “community, social, and civic life.” Previous studies which have reported on the goals of PwPD [11, 13, 14] have been focused on cognitive rehabilitation, whereas this trial had a broad occupation-based rehabilitation focus. Participants in this study could select any goals based on challenges they perceived were most important for them to address, which could have been related to cognitive changes, motor changes or other PD-related impairments. Despite these differences in the studies, the goals identified by PwPD were in similar areas. This suggests that PwPD are having similar daily living challenges in areas of life at home and in the community, reinforcing the importance of access to support and community-based rehabilitation services to address activity and participation restrictions.

In this study “self-care” goals constituted the largest proportion of all goals, predominately in the “managing diet and fitness” chapter. Goals under the “managing diet and fitness” ICF chapter accounted for almost 20% of the goals set by participants and 100% of these goals related to commencing and/or maintaining exercise programs. 85% of the sample set a goal of this nature highlighting the prevalence. Exercise is frequently recommended as a nonpharmacological intervention to protect functioning, manage motor and nonmotor symptoms, and slow disease progression [47]. The predominance of exercise goals in this study indicate that PwPD understand the importance of exercise and aspire to exercise, which is aligned with previous literature [48], but that they may need additional supports from their healthcare teams to establish and maintain regular routines to manage their exercise participation around all PD symptoms.

This study makes a unique contribution to the literature by exploring the underlying impairments related to goal selection in addition to reporting on the nature of goals. We found that approximately 73% of the goals in this study were related to impairments in “mental functions,” compared to 26% which were related to “neuromusculoskeletal and movement-related functions.” Research shows that even subtle cognitive impairments influence performance of daily activities [49] which could explain this finding. In this study, participants perceived that they were experiencing cognitive changes impacting on daily life according to the PDQ, which may indicate that they were wanting to address it early and were attuned to subtle changes in their own cognitive functioning that were not identified on the standardised screening assessment (ACE-III). The smaller proportion “neuromusculoskeletal and movement-related functions” goals could also be because all participants were receiving pharmacological intervention for motor symptom management but that pharmacological treatments are less available or effective for “mental functions” symptoms like executive dysfunction and fatigue [50]. These findings further challenge the view that motor symptoms are the primary problem in PwPD, as the results suggest that nonmotor impairments were more impactful on daily functioning in this sample.

In this study we used detailed therapist field notes describing DPA findings, which provided insights into specific impairments which were impacting on goal-related performance. This provided a novel perspective into the daily functioning of PwPD and was useful in illuminating why PwPD have difficulty in performance and participation. This information was necessary to understand how PD impairments impacted on performance, as this could not be determined from the standardised, objective testing conducted alone (i.e. ACE-III). This finding reinforces the importance of multifaceted, early and comprehensive assessment of neuropsychological functioning, ideally supplemented with assessment of goal-related performance and objective assessments of PD impairments and functioning, to inform treatment planning [51].

Comparing the nature of goals and related underlying impairments by goal priority provides insights into the motivations, perceived burden of PD symptoms, and priorities of PwPD for rehabilitation. This aspect of our study is also a unique contribution in the literature. We found that regardless of priority level, goals were primarily related to impairments in the “mental functions” ICF chapter. This finding is consistent with previous literature reporting that non-motor difficulties are perceived as more restrictive than motor symptoms in daily life [52, 53]. Participants' priority goals also represented their motivation to improve or maintain their performance in everyday activities such as exercise, home organisation, time management, and socialisation. These findings offer insights into the perceived needs of PwPD living in the community which may inform the design and content of rehabilitation programs for PwPD.

This study had a small and culturally homogeneous sample of community-dwelling individuals, who were independent in ADLs, with high levels of education, and therefore the findings may not be representative of the wider population of PwPD. Given the extensive interplay and complexity of symptoms in PD, we cannot be certain that problems with goal-related performance were solely underpinned by one symptom of PD; however, the prolonged engagement of the treating therapist with the participants, along with the structured DPA process, allowed comprehensive assessment of the primary underlying impairment and elicited a thorough understanding of the primary issue contributing to the goal-related problem. Whilst there is the potential for bias in the results due to the first authors' involvement in conducting the COPM, GAS, DPA, CO-OP intervention, data analysis, interpretation, and writing this paper, strategies to mitigate bias included use of an objective and structured coding system, the use of two independent coders not involved in goal setting and consensus meetings.

## 5. Conclusion

This study revealed the complex and diverse challenges that PwPD experience in managing their daily lives. By increasing awareness regarding the goals of PwPD, we hope that important clinical and societal outcomes will result, such as novel intervention approaches that meet identified needs and priorities. These findings indicate that nonmotor symptoms of executive dysfunction and amotivation have a significant impact on performance of, and participation in, a wide array of everyday activities of importance to PwPD, potentially offering guidance to inform the rehabilitation approaches required to meet these needs comprehensively.

## Figures and Tables

**Table 1:** DPA examples.

Example 1	Example 2
One participant's goal was to improve their adherence to their PD home exercise program. They had purchased the program, had completed initial sessions with an exercise physiologist and were motivated to improve their frequency and consistency in completing the home program. The participant described organisational problems as the primary problem during guided discovery, whereby they intended to complete the home program each day but that other daily demands took precedence, and their exercises didn't get done. The participant developed a strategy that involved pairing their home exercise program with another activity that occurred daily (eating lunch) and sharing digital tracking of their activity with family members (active minutes) to increase accountability. The strategy addressed planning and organisation problems and was effective in enabling task performance	One participant's goal was to carry a tray of drinks, without spilling. They knew how to perform this activity and were adequately motivated for task performance. Observation of their performance, and discussions during guided discovery, indicated that tremor, anxiety and poor self-efficacy were contributing to ineffective performance. Several participant strategies were trialled during DPA including: Using melamine cups instead of porcelain cups and reducing the volume of liquid in cups to overcome anticipatory anxiety; and using nonslip matting on the tray to minimize cups slipping with tremor. The most effective strategy involved positioning of the participant's thumb (bilateral interphalangeal extension), which reduced the intention tremor when carrying the tray

**Table 2:** Participant characteristics.

Age, years (SD)	68.10 (8.1)
Sex, *n* (%)	
Male	10 (45.5)
Female	12 (54.5)
Duration of PD, years (SD)	5.7 (3.5)
Hoehn & Yahr Stage, *n*	
Stage 1	2
Stage 1.5	5
Stage 2	3
Stage 2.5	3
Stage 3	8
Stage 4	1
ACE-III, mean (SD)	94.5 (5.4)
NEADL, mean (SD)	18.4 (2.9)
PDQ, mean (SD)	27.33 (10.15)
NART, mean (SD)	117.4 (5.4)
Education level, highest attainment, *n*	
Junior certificate	4
High school certificate	2
Trade certificate	2
Diploma	1
Bachelor's degree	6
Masters degree	3
Other postgraduate degree	4
Work status, *n*	
Working full time	3
Not working due to health	5
Retired	13
Semi-retired	1

ACE-III = Addenbrooke's cognitive evaluation III; NEADL = Nottingham extended living activities of daily living scale; PDQ = perceived deficits questionnaire; NART = national adult reading test.

**Table 3:** Number and percentage of goals classified by nature of goals according to ICF activity and participation chapters.

ICF activity and participation first and second level chapter codes	*n* (%)	ICF description of chapter and example GAS goals
Self-care	20 (22.7)	This chapter is about caring for oneself, washing and drying oneself, caring for one's body and body parts, dressing, eating and drinking, and looking after one's health
d5701 managing diet and fitness	17 (19.3)	Increase participation in home exercise programs
d540 dressing	2 (2.3)	Independently dress in clothing desired to wear instead of what can manage
d5702 maintaining one's health	1 (1.1)	Reduce missed dose or late dose incidences to 3 times per week
General tasks and demands	18 (20.5)	This chapter is about general aspects of carrying out single or multiple tasks, organising routines and handling stress. These items can be used in conjunction with more specific tasks or actions to identify the underlying features of the execution of tasks under different circumstances
d2302 completing the daily routine	7 (8.0)	Use a list to prioritise tasks and completes 85–89% of tasks on list
d220 undertaking multiple tasks	2 (2.3)	Sort, scan, and upload photos of children to create photobooks
d230 carrying out daily routine	2 (2.3)	Improve planning with washing and dressing related to going out and being on time
d2303 managing one's own activity level	2 (2.3)	Fatigue management with scheduling and activity completion
d2100 undertaking a simple task	1 (1.1)	Decrease time taken to send emails on smart phone
d2304 adapting to changes in daily routine	1 (1.1)	Reduce the incidence and impact of tremors at work
d240 handling stress and other psychological demands	1 (1.1)	Improve ability to manage emotions in loud environments
d2400 handling responsibilities	1 (1.1)	Reduce incidence of forgetting appointments
d2401 handling stress	1 (1.1)	Reduce incidence of forgetting things when stressed
Domestic life	13 (14.8)	This chapter is about carrying out domestic and everyday actions and tasks. Areas of domestic life include caring for one's belongings and space, acquiring food, clothing and other necessities, household cleaning and repairing, caring for personal and other household objects, and assisting others
d640 doing housework	8 (9.1)	Use pressure cleaner at home
d650 caring for household objects	4 (4.5)	Refurbish adirondack chairs
d630 preparing meals	1 (1.1)	Increase independence and ease with vegetable preparation (peeling and cutting)
Community, social and civic life	11 (12.5)	This chapter is about the actions and tasks required to engage in organised social life outside the family, in community, social and civic areas of life
d920 recreation and leisure	8 (9.1)	Increase participation in creative and leisure activities
d9100 informal associations	2 (2.3)	Engage in social and leisure activities with others 3-4 days per week
d950 political life and citizenship	1 (1.1)	Engage in social and volunteer activities 3-4 days per week
Mobility	11 (12.5)	This chapter is about moving by changing body position or location or by transferring from one place to another, by carrying, moving or manipulating objects, by walking, running or climbing, and by using various forms of transportation
d420 transferring oneself	5 (5.7)	Increase independence with sit to stand transfers
d415 maintaining body position	1 (1.1)	Reduce incidence of falls
d429 changing and maintaining body position, other specified and unspecified	1 (1.1)	Experience decreased pain/discomfort in recliner chair in the evenings
d4301 carrying in the hands	1 (1.1)	Reduction of spilling when carrying drinks
d4400 picking up	1 (1.1)	Improve ease and speed with getting keys from handbag
d4401 grasping	1 (1.1)	Improve ease and speed opening packets and jars
d4452 reaching	1 (1.1)	Improve ability to access things in low cupboards
Communication	8 (9.1)	This chapter is about general and specific features of communicating by language, signs and symbols, including receiving and producing messages, carrying on conversations, and using communication devices and techniques
d345 writing messages	6 (6.8)	Improve touch typing speed
d3501 sustaining a conversation	2 (2.3)	Maintain engagement and participates in conversation
Learning and applying knowledge	4 (4.5)	This chapter is about learning, applying the knowledge that is learned, thinking, solving problems, and making decisions
d140 learning to read	1(1.1)	Improve fluency (speed, accuracy, and expression) when reading aloud
d155 acquiring skills	1 (1.1)	Reduce incidence of forgetting names that I should know
d1601 focusing attention on the environment	1 (1.1)	Recover from and reduce frustration from distractions
d163 thinking	1 (1.1)	Minimise incidences of forgetfulness
Interpersonal interactions and relationships	2 (2.3)	This chapter is about carrying out the actions and tasks required for basic and complex interactions with people (strangers, friends, relatives, family members and lovers) in a contextually and socially appropriate manner
d7502 informal relationships with acquaintances	1 (1.1)	Reduce tremor anxiety in social situations with others
d799 interpersonal interactions and relationships, unspecified	1 (1.1)	Participate in 2 meaningful social interactions daily
Major life areas	1 (1.1)	This chapter is about carrying out the tasks and actions required to engage in education, work and employment and to conduct economic transactions
d845 acquiring, keeping and terminating a job	1 (1.1)	Improve planning and scheduling of work activities to manage workload

**Table 4:** Number and percentage of goals classified by primary underlying impairment according to ICF Body Functions chapters.

ICF body functions first and second level chapter codes	*n* (%)	ICF description of chapter and example GAS goals
Mental functions	64 (72.7)	This chapter is about the functions of the brain: both global mental functions, such as consciousness, energy and drive, and specific mental functions, such as memory, language, and calculation mental functions
b1641 organisation and planning	23 (26.1)	Bedroom is mostly organised
b1642 time management	16 (18.2)	Completes planned tasks or jobs scheduled for the day on 3-4 days per week
b1301 motivation	10 (11.4)	Engage in social and volunteer activities 3-4 days per week
b1443 working memory	5 (5.7)	Reduce missed dose or late dose to 3 times per week
b1521 regulation of emotion	3 (3.4)	Recover from and reduce frustration from distractions
b1300 energy level	3 (3.4)	Increase incidences of going out and/or completing heavy home chores per week
b1671 expression of language	3 (3.4)	Reduce word finding problems, increase participation in conversation
b1266 confidence	1 (1.1)	Resume travel for leisure (increase confidence)
Neuromusculoskeletal and movement-related functions	23 (26.1)	This chapter is about the functions of movement and mobility, including functions of joints, bones, reflexes, and muscles
b7602 coordination of voluntary movements	11 (12.5)	Improve ease and independence with moving in bed
b7601 control of complex voluntary movements	7 (7.9)	Handwriting will increase in legibility
b7651 tremor	4 (4.5)	Manage tremor when socialising
b755 involuntary movement reaction functions	1 (1.1)	Reduce number of falls
Sensory functions and pain	1 (1.1)	This chapter is about the functions of the senses, seeing, hearing, tasting, and so on, as well as the sensation of pain
b2802 pain in multiple body parts	1 (1.1)	Experiences decreased pain/discomfort in the evenings

**Table 5:** Number and percentage of goals classified by ICF body functions chapter for highest to lowest priority goals.

ICF body functions chapters, *n* (%)	Goal 1	Goal 2	Goal 3	Goal 4
Mental functions	15 (68.2)	19 (86.4)	17 (77.3)	14 (63.6)
Neuromusculoskeletal and movement related functions	6 (27.3)	3 (13.6)	5 (22.7)	8 (36.4)
Sensory functions and pain	1 (4.5)	—	—	—

**Table 6:** Number and percentage of goals classified by ICF activity and participation chapter for highest to lowest priority goals.

ICF activity and participation chapters, *n* (%)	Goal 1	Goal 2	Goal 3	Goal 4
Self-care	6 (27.3)	7 (31.8)	2 (9.1)	5 (22.7)
General tasks and demands	5 (22.7)	4 (18.2)	6 (27.3)	2 (9.1)
Community social and civic life	1 (4.5)	5 (22.7)	4 (18.2)	1 (4.5)
Mobility	4 (18.2)	2 (9.1)	4 (18.2)	1 (4.5)
Domestic life	3 (13.6)	1 (4.5)	1 (4.5)	8 (36.4)
Learning and applying knowledge	1 (4.5)	1 (4.5)	—	2 (9.1)
Communication	1 (4.5)	1 (4.5)	3 (13.6)	3 (13.6)
Interpersonal interactions and relationships	1 (4.5)	—	2 (9.1)	—
Major life areas	—	1 (4.5)	—	—

## Data Availability

The data that support the findings of this study are available on request from the corresponding author. The data are not publicly available due to restrictions of ethical clearances.
